# Networked information technologies and patient safety: a protocol for a realist synthesis

**DOI:** 10.1186/s13643-019-1223-1

**Published:** 2019-12-05

**Authors:** Justin Keen, Joanne Greenhalgh, Rebecca Randell, Peter Gardner, Justin Waring, Roberta Longo, Jon Fistein, Maysam Abdulwahid, Natalie King, Judy Wright

**Affiliations:** 1 Leeds Institute of Health Sciences, University of Leeds, Worsley Building, Clarendon Way, Leeds, LS2 9NL England; 2 School of Sociology and Social Policy, University of Leeds, Leeds, England; 3 School of Healthcare, University of Leeds, Leeds, England; 4 School of Psychology, University of Leeds, Leeds, England; 5 Health Services Management Centre, University of Birmingham, Birmingham, England

**Keywords:** Patient safety, Information technology, Systems integration, Health information exchange, Health services research, Systematic review, Realist synthesis

## Abstract

**Background:**

There is a widespread belief that information technologies will improve diagnosis, treatment and care. Evidence about their effectiveness in health care is, however, mixed. It is not clear why this is the case, given the remarkable advances in hardware and software over the last 20 years. This review focuses on interoperable information technologies, which governments are currently advocating and funding. These link organisations across a health economy, with a view to enabling health and care professionals to coordinate their work with one another and to access patient data wherever it is stored. Given the mixed evidence about information technologies in general, and current policies and funding, there is a need to establish the value of investments in this class of system. The aim of this review is to establish how, why and in what circumstances interoperable systems affect patient safety.

**Methods:**

A realist synthesis will be undertaken, to understand how and why inter-organisational systems reduce patients’ clinical risks, or fail to do so. The review will follow the steps in most published realist syntheses, including (1) clarifying the scope of the review and identifying candidate programme and mid-range theories to evaluate, (2) searching for evidence, (3) appraising primary studies in terms of their rigour and relevance and extracting evidence, (4) synthesising evidence, (5) identifying recommendations, based on assessment of the extent to which findings can be generalised to other settings.

**Discussion:**

The findings of this realist synthesis will shed light on how and why an important class of systems, that span organisations in a health economy, will contribute to changes in patients’ clinical risks. We anticipate that the findings will be generalizable, in two ways. First, a refined mid-range theory will contribute to our understanding of the underlying mechanisms that, for a range of information technologies, lead to changes in clinical practices and hence patients’ risks (or not). Second, many governments are funding and implementing cross-organisational IT networks. The findings can inform policies on their design and implementation.

**Systematic review registration:**

PROSPERO CRD42017073004

## Background

There is a widespread belief, particularly among policy makers, that information technologies will improve diagnosis, treatment and care [1]. Evidence about the effectiveness of a range of IT applications is, however, mixed [2–5]. A comprehensive review by Brenner and colleagues covered a number of technologies and focused on safety-related end-points including mortality, infection rates and medication error rates [2]. Twenty-five out of 69 studies included in their review reported a statistically significant positive effect. The other 44 studies reported no or negative effects. More recent systematic reviews report broadly similar findings [6, 7]. Authors stress that evidence is uneven in both coverage and quality, and care needs to be taken in interpreting the findings, but the general picture is clear.

There is, therefore, a need to understand *why* the evidence has, to date, fallen short of expectations. We seek to shed light on the question in this systematic review. It focuses on the effects of a particular class of IT systems, interoperable systems, on patient safety. The term ‘system’ here refers to the combination of technologies and the people who used them. In a well-designed system, technologies are seamlessly integrated into users’ working practices. In poorly designed systems, in contrast, the technologies do not fit easily into users’ working practices, and in the worst cases can make it more difficult to deliver safe treatment and care. The term ‘interoperable’ refers to the ability of any two or more IT systems to exchange data, and for the receiving system to make use of the data. The IT systems of interest in this protocol allow professionals to access patients’ records held in other organisations.

## Policy makers’ assumptions

Many people who live in their own homes, and who are frail or have chronic health problems, need support from a range of professionals. These include general (or family) practitioners, community nurses, therapists, social workers, and planned and emergency hospital services. There is good evidence that treatment and care is often fragmented [8–10]. Linking IT systems across a health economy can, policy makers reason, help to improve the coordination of services. Professionals can, the thinking goes, use the IT networks to communicate with one another, and hence effectively coordinate a patient’s treatment and care. The value of this function might be particularly evident at transition points, for example in a health emergency or at the point of returning from hospital to home. The IT systems can also be designed to enable access to all parts of a patient’s record, so that professionals can search for and locate information wherever it is held. The Obama administration in the USA committed considerable sums to linking hospital, family physician, pharmacy and other IT systems. Similarly, and more recently, the National Health Service in England has emphasised the importance of linking hitherto fragmented IT systems across organisations in local health economies [11, 12].

## Realist synthesis

This protocol describes a realist synthesis, which seeks to understand how and why inter-organisational IT systems reduce patients’ clinical risks, or fail to do so. The realist synthesis method involves opening up the ‘black box’ of events that lie between an intervention and its effects [13, 14]. It does so by identifying programme theories: these are sequences of decisions and actions that capture the *intended* effects of an intervention, and the underlying logic that links them together. A number of initial theories are typically identified.

Literature searches are then designed. It is not usually feasible to identify evidence about all of the sequences in all of the theories. Searches therefore focus on two or three theories, or on key sequences within those theories. Empirical evidence is then identified, assessed and synthesised, in order to evaluate the *actual* steps in the sequences. The evidence can, in addition, lead teams to realise that a programme is effective, but not in the way originally envisaged. A programme theory therefore needs to be reformulated. More searches may then be needed, to evaluate elements of the revised theory. That is, the cycle of programme theory formulation and evaluation can be iterative and—resources permitting—pursued until a settled, evidence-based account has been identified [15].

There are literatures that will enable us to develop and evaluate programme theories [16]. For example, the computer-supported cooperative work literature sits at the intersection of computer science and psychology, and is a source of evidence about the inter-relationships of IT systems and peoples’ working practices. A comprehensive review suggests that we have a reasonable understanding of the design features and organisational settings that are associated with the effective integration of systems into clinical working practices [17]. Similarly, there are literatures on health professionals’ decisions and actions, and their consequences for patients’ clinical risks. In particular, there is a substantial literature based on retrospective analysis of adverse events, both in health care and other sectors [18].

This protocol has been written in accordance with PRISMA-P guidelines [19].

## Methods

We will undertake a realist synthesis. The aim of the study is to establish how, why and in what circumstances networked, inter-organisational IT systems affect patient safety. The objectives of the study are to:

1. Identify initial programme theories and prioritise theories to review;
2. Search systematically for evidence to test and refine the theories;
3. Undertake quality appraisal and use included texts to support, refine or reject programme theories;
4. Synthesise the findings;
5. Disseminate the findings to a range of audiences.

The following sections focus on the first four objectives.

### Stage 1: Identification of programme and mid-range theories

This stage is essentially developmental in nature. The first step in this review will be to construct one or more programme theories, concerning the use of networked IT systems and their effects on patient safety. Mid-range theories, which are usefully thought of as a broader class of theory than programme theories, and which will be used to generalise findings at the end of the review, will also be identified. We will supplement our existing knowledge of candidate theories with literature searches to define and develop them. The programme theories will determine the scope (inclusion and exclusion criteria) for the searches and synthesis in the following stages, which will be used to support, refine or reject each theory examined.

#### Search strategy and information resources

Theories can be explicitly mentioned in research articles and policy documents, or they can be implied in the introductory or discussion sections of documents. They can also be found in commentaries and opinion pieces. We will use conventional literature searching using free text words, synonyms and subject index terms, and some CLUSTER searching techniques (identifying a few key relevant studies and finding further relevant studies via forwards and backwards citation searches, author searches, searching for reports of a particular project) [20]. Using this combined approach, we aim to identify literature that leads us to theories or fragments of theories that can be used to construct programme and mid-range theories.

We anticipate that three searches will supplement the policy documents and academic literature already known to the project team. MEDLINE (1946–present) and EMBASE (1947–present) will be searched as a core set of databases for all of the theory generating searches.

1. *Background search of systematic reviews*. We will search for systematic reviews that link IT systems and patient safety. This will identify any reviews on this topic that have been published since our scoping review, undertaken before the start of the project. The reviews may describe programme theories or fragments in their introduction or discussion sections, providing insights into the sequences of events linking the intervention and outcomes. We will search the core databases plus the Cochrane Database of Systematic Reviews, the Epistemonikos database and Health Systems Evidence (McMaster University). An example MEDLINE search strategy containing a full set of search terms is available in the Additional file 1 (Search 1).
2. *Policies, opinion pieces and research reports*. We will search the core databases plus Health Management Information Consortium (1983–present) and Web of Science - Science Citation Index (1990–present) for policy documents, opinion pieces (e.g. editorials) and reports describing leading theories about the relationships between IT systems and patient safety (Additional file 1 Search 2). We will also undertake Google searches to locate reports on key policies, e.g. about the Health Information Technology for Economic and Clinical Health (HITECH) Act 2009, a major US initiative promoting the implementation of cross-organisational IT networks.
3. *Author search and Citation search*. We will search for reports and articles authored by influential commentators in the core databases plus Health Management Information Consortium (1983–present), Web of Science - Science Citation Index (1990–present), Google Scholar and Scopus (1823–present). Literature by David Bates, the most cited author in the health informatics literature, and Robert Wachter, the author of an influential report on IT in the NHS in England, will be searched (Additional file 1 Search 3). Citation searching may be required due to the iterative nature of developing searches for a realist synthesis.

#### Inclusion, analysis and synthesis

The records identified in the searches will be saved and managed in an EndNote library. Details of all search activities (databases, websites, date of search, number of records found, search strategies) will be recorded in a timeline spreadsheet. The inclusion criteria for the three searches will be:

- IT networks that link two or more organisations outside (but possibly including) hospitals;
- IT networks that support direct treatment and care;
- Arguments that identify relationships between IT networks and patient safety;
- Published in the English language between 2000 and 2018.

Non-English language articles will not be included. The project does not have sufficient resources to translate the full text of articles that may be relevant. In conventional systematic reviews, it is not necessary to translate whole papers, as it is only necessary to identify defined data. In this review, however, we will be looking for data that might occur anywhere in a paper, and a full translation would be needed.

The exclusion criteria will be for studies that:

- Describe hospital-only IT systems;
- Describe systems that do not link two or more distinct services;
- Focus on IT systems that support secondary uses of data, e.g for service planning, research;
- are published before 2000;
- are published in languages other than English.

Two reviewers (MA and JK) will first independently screen the titles and abstracts of the records for relevance and then assess full-text reports. Any discrepancies will be solved by discussion, and if needed consultation with a third author (JG or RR). Data extraction forms will be developed to capture basic details of studies—authors, publication year, etc.—and passages on theories and theory fragments.

The selected studies will be used to develop visual representations of programme theories, with accompanying text that explains the reasoning that underpins those theories. Experience gained in earlier reviews suggests that there are likely to be several programme theories at this point, and that some of them will be partial, in the sense that the chains of reasoning are ‘high level’ and not fully articulated, or only cover some of the steps linking networked information technologies and patient safety. Where available, claims about the reasons why programmes succeed or fail in practice will be used to annotate the representations.

The programme theories will be used as the basis for consultation with three groups of stakeholders—policy makers, senior IT managers and frontline clinicians. We will use the nominal group technique, which has been used in a previous realist study [21]. At the nominal group meetings participants will be asked to comment critically on the programme theories, on the basis of their knowledge and experience. They will also be asked to develop and then prioritise theories, or particular chains of reasoning within theories, for further study. The prioritisation will take into account the potential to provide learning for the NHS, and the types of networked systems that NHS organisations are implementing. The groups will be re-convened for consultation by email at the end of stage 4 (see below).

The outputs of the three groups will be further reviewed by a patient and public involvement (PPI) group, and discussed with our project steering group. Following these meetings, we will decide on the programme theories, and key elements of those theories, that we will explore in depth in stages 2–4.

### Stage 2: Systematic search for evidence

The next stage of the review is a search for empirical studies to test and refine the leading programme theories identified in stage 1. The initial searches will be designed to identify evidence about the steps in the chains of reasoning in each theory. Literatures often focus on one or other section in a chain of reasoning, and as a result, individual searches will often focus on sections rather than a whole programme theory [22]. We will undertake searches using resources which span health and computing literatures including—but not limited to—MEDLINE (1946–present), EMBASE (1947–present), Web of Science Core Collection (1900–present) and INSPEC (1896–present).

We anticipate hand searching papers from leading conferences which are not indexed, for example Software Engineering in Healthcare workshops papers, from the International Conference on Software Engineering. The search strategies for identifying empirical evidence for programme theories can only be fully developed once the programme theories are agreed. However, we anticipate they will contain search concepts for an aspect of patient safety such as medication reconciliation, inter-organisational IT networks and evaluative studies.

The search results will be reviewed in stage 3 (evidence review, see below) and further searches will develop iteratively to follow lines of enquiry. Initial searches may not identify empirical evidence that supports or rejects a programme theory. If that happens, the search will be re-designed to capture empirical evidence that may be found in a different discipline or information resource. For example, we could extend the scope of our searches to look for evidence about other IT applications, including hospital-based systems, and/or extend the scope of the populations of interest. As in stage 1, these searches may use CLUSTER search techniques as an efficient method for finding relevant papers [20].

The results of the electronic searches and all references that are retrieved for stage 2 will be kept in the same EndNote library as those found during stage 1. Details of all search activities during stage 2 will be recorded in the timeline spreadsheet.

### Stage 3: Evidence review and quality appraisal

Titles and abstracts of records identified, and the full-text papers selected in stage 2, will be independently screened by two reviewers (MA and JK) to identify those which contain evidence that sheds light on one or more elements of the programme theories identified in stage 1. The RAMESES I guidance states that:

An appraisal of the contribution of any section of data (within a document) should be made on two criteria:

• *Relevance* – whether it can contribute to theory building and/or testing; and

• *Rigour* – whether the method used to generate that particular piece of data is credible and trustworthy [14].

We will follow Rycroft-Malone and colleagues in developing criteria for judging relevance [23]. We will, further, use the mid-range theory developed in stage 1 to refine the criteria. Rigour refers to the requirement for an investigation to be of sufficient standard *within type*, whether that is a process evaluation, an ethnography or other type of study [14].

The empirical data for supporting and/or refuting programme theories will be extracted from the included studies. It is anticipated that a significant proportion of the evidence will be in the form of narrative data and will accordingly be copied into Word files. To maximise accuracy and transparency, a proportion of data extraction will be performed independently by two members of the research team.

### Stage 4: Synthesis

Synthesis involves two distinct, but linked, activities. In the first, the empirical evidence identified in stages 2 and 3 will be used to evaluate the programme theories developed in stage 1. In the most straightforward case, the evidence will support the chains of reasoning in one programme theory and serve to reject alternative or competing theories. Less straightforwardly, the evidence might provide support for one part of a programme theory and adverse evidence for another part of the same theory. Or, it might not ‘fit’ a programme theory, neither supporting nor undermining it. Both instances suggest that there may be a problem with the programme theory itself and will lead us to refine it, to achieve a better fit between evidence and theory. On the basis of experience of earlier realist syntheses, we expect that at least one of the selected theories—or theory fragments—will be reasonably well supported by empirical evidence, and at least one will not be not supported.

Second, when a settled programme theory or theories have been produced, they will be interpreted in the broader context of the mid-range theory. This involves abduction, where inferences that lead to the best available explanation are identified. The details of the abductive reasoning processes vary from review to review, but a key point is that it involves inter-play between situation-specific programme theories and broader mid-range theory [24]. In the simplest case, the (now evidence-based) programme theories will be consistent with the mid-range theory. If we find that a programme theory holds across a number of settings (e.g. different combinations of health services and/or different patient groups), this will increase our confidence in it. Alternatively, evidence or argument (or both) may point in different directions, and the wider project team will use the mid-range theory to ‘adjudicate’ between contending programme theories.

#### Nominal group email consultation

The nominal groups will be re-convened for email consultation. We will summarise our findings to this point, including our provisional syntheses, and present them to the groups. They will be asked to comment on the findings, including whether any of the theories can be rejected, and whether any further searches are merited. The PPI group will also meet at the end of this stage and review the findings and interpretations of the three nominal groups. We will refine our interpretations on the basis of the comments of all four groups.

## Discussion

Policy makers in many countries believe that IT systems can contribute to safer patient care. Interoperable systems are currently being promoted by governments, and funding made available for their development, in many countries. As noted above, though, the empirical evidence about this belief is mixed, and it is not clear why this is the case. We are using the realist synthesis method because we believe that it will shed light on how and why interoperable systems contribute to reductions in patients’ clinical risks, and hence help to explain why the evidence is so mixed.

As with any evidence synthesis, there are risks and limitations associated with the method. The most obvious risk, in common with other review methods that focus on effectiveness, is that we are not able to identify high-quality effectiveness evidence. This will necessarily limit the extent to which we are able to explain ‘what works’ when interoperable systems are deployed. Another significant risk is the flip side of a potential strength. The explicit inclusion of theory in the method means that the basis for interpreting the available evidence is clear. But, there must be a risk that—however much care is taken—a sub-optimal theoretical framework will be used, and valuable insights foregone. If we are able to mitigate these risks in the course of the review, though, it should produce two main outcomes. First, the refined mid-range theory will contribute to our understanding of the underlying mechanisms that lead to changes in clinical practices and hence patients’ risks (or not). Second, many governments are funding and implementing cross-organisational IT networks. The findings can be used to inform policies on their design and implementation.

## Supplementary information

**Additional file 1.** Search strategies to identify reports for generating theory.

## Data Availability

This is a protocol for a systematic review. We will make all searches, and the results of those searches, available publicly when the review is completed. This will, again, require clearance by NIHR—but we do not anticipate any problems, given the nature of the study.

## References

[1] Agboola SO, Bates DW, Kvedar JC (2016). Digital health and patient safety. JAMA.

[2] Brenner SK (2016). Effects of health information technology on patient outcomes: a systematic review. J Am Med Inform Assoc.

[3] Black AD (2011). The impact of eHealth on the quality and safety of health care: a systematic overview. PLoS Med.

[4] Kruse CS, Beane A (2018). Health information technology continues to show positive effect on medical outcomes: systematic review. J Med Internet Res.

[5] Chaudhry B (2006). Systematic review: impact of health information technology on quality, efficiency, and costs of medical care. Ann Intern Med.

[6] Feldman SS, Buchalter S, Hayes LW (2018). Health information technology in healthcare quality and patient safety: literature review. JMIR Med Inform.

[7] Garavand A (2016). Factors influencing the adoption of health information technologies: a systematic review. Electron Physician.

[8] O’Hara JK, Aase K, Waring J (2019). Scaffolding our systems? Patients and families ‘reaching in’ as a source of healthcare resilience. BMJ Qual Saf.

[9] Care Quality Commission, Building bridges, breaking barriers. Newcastle upon Tyne: CQC, 2017.

[10] Leigh Johnston DR, Dorrans S, Dussin L. A.T. Barker, Integration 2020: Scoping research. p. 2017.

[11] Jacob JA (2015). On the road to interoperability, public and private organizations work to connect health care data. JAMA.

[12] Bates DW (2015). Health information technology and care coordination: the next big opportunity for informatics?. Yearb Med Inform.

[13] Pawson R (2006). Evidence Based Policy: A Realist Perspective.

[14] Wong G (2014). Development of methodological guidance, publication standards and training materials for realist and meta-narrative reviews: the RAMESES (Realist And Meta-narrative Evidence Syntheses – Evolving Standards) project. Health Serv Del Res.

[15] Pawson R (2013). The Science of Evaluation: A Realist Manifesto.

[16] Pollock N, Williams R (2009). Software and organisations. The biography of the enterprise-wide system or how SAP conquered the world.

[17] Fitzpatrick G, Ellingsen G (2013). A review of 25 years of CSCW research in healthcare: contributions, challenges and future agendas. Comput Supp Coop Work (CSCW).

[18] Vincent, C. and R. Amalberti, Safety strategies in hospitals, in Safer Healthcare. SpringerOpen, 2016, p. 73-91.

[19] Moher D (2015). Preferred reporting items for systematic review and meta-analysis protocols (PRISMA-P) 2015 statement. Syst Rev.

[20] Booth A (2013). Towards a methodology for cluster searching to provide conceptual and contextual “richness” for systematic reviews of complex interventions: case study (CLUSTER). BMC Med Res Methodol.

[21] Tolson D (2007). Developing a managed clinical network in palliative care: a realistic evaluation. Int J Nurs Stud.

[22] Ball SL, Greenhalgh J, Roland M (2016). Referral management centres as a means of reducing outpatients attendances: how do they work and what influences successful implementation and perceived effectiveness?. BMC Fam Pract.

[23] Rycroft-Malone J (2014). Improving skills and care standards in the support workforce for older people: a realist review. BMJ Open.

[24] Pawson R (2002). Evidence and policy and naming and shaming. Policy Stud.

