# Supplementary material for: Networked information technologies and patient safety: a protocol for a realist synthesis
Source: Syst Rev. 2019 Dec 5;8:307. doi: 10.1186/s13643-019-1223-1 (PMC6896666; doi:10.1186/s13643-019-1223-1)
Supplement: Supplementary file 1 — Additional file 1. Search strategies to identify reports for generating theory. [file 13643_2019_1223_MOESM1_ESM.docx]

Additional file

**Search strategies to identify reports for generating theory**

**Search 1 Background search of systematic reviews**

Ovid MEDLINE(R) <1946 to January Week 4 2018>

Search Strategy:

--------------------------------------------------------------------------------

1 "Safety Management"/ and (outpatient* or patient*).tw. (7025)

2 "Medical Errors"/ and (outpatient* or patient*).tw. (7345)

3 patient safety/ (13530)

4 ((patient or patients or outpatient*) adj2 (safety or harm* or adverse*)).tw. (31423)

5 *risk management/ (8546)

6 or/1-5 [Patient Safety] (53147)

7 medical informatics/ (10307)

8 health information exchange/ (536)

9 medical informatics applications/ (2302)

10 medical informatics computing/ or nursing informatics/ or public health informatics/ (3147)

11 exp *information systems/ (96193)

12 exp *decision making, computer-assisted/ (87416)

13 ("Information technolog*" or computer*).ti. (64585)

14 *technology/ or *biomedical technology/ (8062)

15 exp *Computer Systems/ (84260)

16 or/7-15 [IT systems] (303456)

17 6 and 16 (2990)

18 limit 17 to "reviews (maximizes specificity)" (72)

**Search 2 Policies, opinion pieces and research reports**

Database: Ovid MEDLINE(R) <1946 to February Week 5 2018>

Search Strategy:

--------------------------------------------------------------------------------

1 (policy or policies or guideline* or recommendation* or position).ti. (157471)

2 guideline/ or practice guideline/ (29721)

3 policy/ or public policy/ or exp health policy/ (126430)

4 (theor* or concep* or logic).ti. (140361)

5 ((theor* or concep* or logic) adj (framework* or model* or analy* or evaluat*)).ab. (44188)

6 or/1-5 [Policy, Guideline or overt Theory] (445038)

7 Comment/ (664894)

8 Letter/ (926791)

9 Editorial/ (405400)

10 news/ or newspaper article/ (190273)

11 "Comment on".ti. (16286)

12 (letter* adj3 editor*).ti. (6376)

13 opinion*.ti. (11812)

14 (view or views).ti. (43579)

15 or/7-14 [Discussion papers Hidden Theory] (1710573)

16 6 or 15 [Theory Search] (2103940)

17 "Safety Management"/ and (outpatient* or patient*).tw. (7050)

18 "Medical Errors"/ and (outpatient* or patient*).tw. (7399)

19 patient safety/ (13787)

20 ((patient or patients or outpatient*) adj2 (safety or harm* or adverse*)).tw. (31734)

21 *risk management/ (8572)

22 or/17-21 [Patient Safety] (53654)

23 Health Information Exchange/ (562)

24 ((health information or medical information or clinical information) adj5 (exchang* or shar* or network*)).tw,kw. (2012)

25 (patient information adj5 (exchang* or shar* or network*)).tw,kw. (198)

26 23 or 24 or 25 (2551)

27 exp Medical Records/ (132774)

28 information systems/ or decision support systems, clinical/ or health information systems/ or exp management information systems/ (67432)

29 medical informatics/ or medical informatics applications/ or medical informatics computing/ or nursing informatics/ or public health informatics/ (15531)

30 exp Decision Making, Computer-Assisted/ (124934)

31 (electronic adj3 record*).tw. (20676)

32 ((health or medical or clinical) adj5 (information or record*)).tw. (190180)

33 Computer Systems/ (12550)

34 or/27-33 [health records] (484060)

35 community network/ (6385)

36 computer communication networks/ or local area networks/ (13961)

37 information dissemination/ (14049)

38 (network* or exchange* or data shar*).tw. (492792)

39 (information adj3 (shar* or distribut* or disseminat*)).tw. (12562)

40 (record* adj3 (shar* or distribut* or disseminat*)).tw. (2196)

41 or/35-40 [networking systems] (529805)

42 41 and 34 (28255)

43 42 or 26 [Networked IT systems] (28641)

44 43 and 22 [safety in networked systems] (468)

45 16 and 44 [Theory & Opinion networked IT safety systems] (47)

**Search 3 Author search and Citation search example**

Database: Ovid MEDLINE(R) <1946 to February Week 5 2018>

Search Strategy:

--------------------------------------------------------------------------------

1 wachter rm.au. (232)

2 wachter.ti,ab,kw. (45)

3 1 or 2 (277)

4 limit 3 to yr="2015 -Current" (18)
